# Supplementary figures and images for: Chemical composition, nutritional profile and in vivo antioxidant properties of the cultivated mushroom Coprinus comatus
Source: R Soc Open Sci. 2020 Sep 2;7(9):200900. doi: 10.1098/rsos.200900 (PMC7540769; doi:10.1098/rsos.200900)

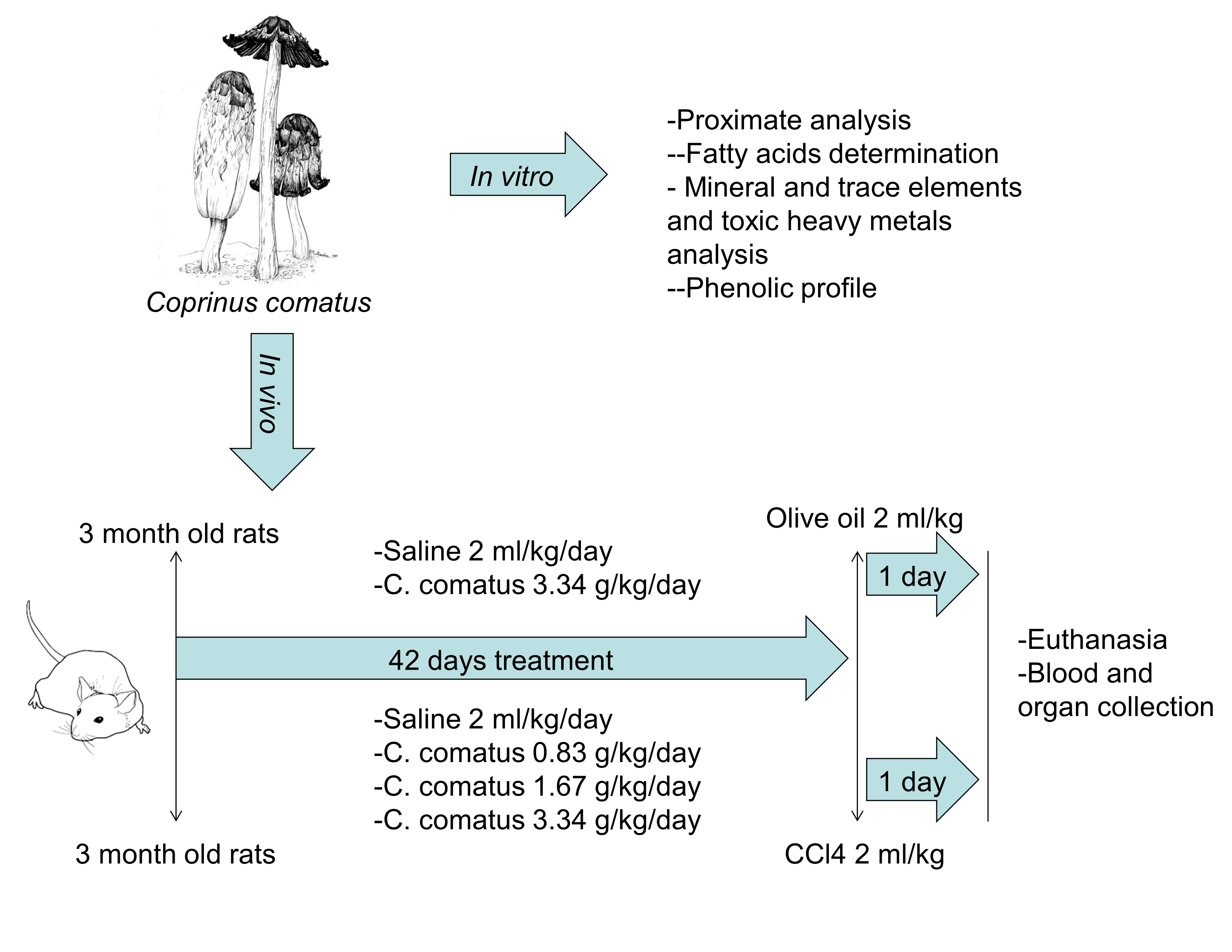

Supplement: Experimental overview [file rsos200900supp1.tif]
